# Glycosylation and methylation in the biosynthesis of isoflavonoids in *Pueraria lobata*

**DOI:** 10.3389/fpls.2023.1330586

**Published:** 2023-12-15

**Authors:** Changfu Li, Yansheng Zhang

**Affiliations:** Shanghai Key Laboratory of Bio-Energy Crops, School of Life Sciences, Shanghai University, Shanghai, China

**Keywords:** glycosylation, methylation, isoflavonoid, pueraria, biosynthesis

## Abstract

The pathway for forming isoflavonoid skeletal structure is primarily restricted to the Leguminosae family. Subsequent decorations on the compound backbone by tailoring enzymes would change their biological and medicinal properties. *Pueraria lobata* is a leguminous plant, and as a traditional Chinese medicine its roots have been ascribed a number of pharmacological activities. Glycosylation and methylation are the main modifying processes in isoflavonoid metabolism in *P. lobata* roots, resulting in the accumulation of unique glycosylated and methylated end isoflavonoid compounds. For instance, daidzein 8-*C*-glucoside (i.e., puerarin) and puerarin derivatives are produced only by the *Pueraria* genus. Puerarin has been established as a clinical drug for curing cardiovascular diseases. To better understand the characteristic isoflavonoid metabolism in *P. lobata*, this review attempts to summarize the research progress made with understanding the main glycosylation and methylation of isoflavonoids in *P. lobata* and their biosynthetic enzymes.

## Introduction

**Figure 1:**
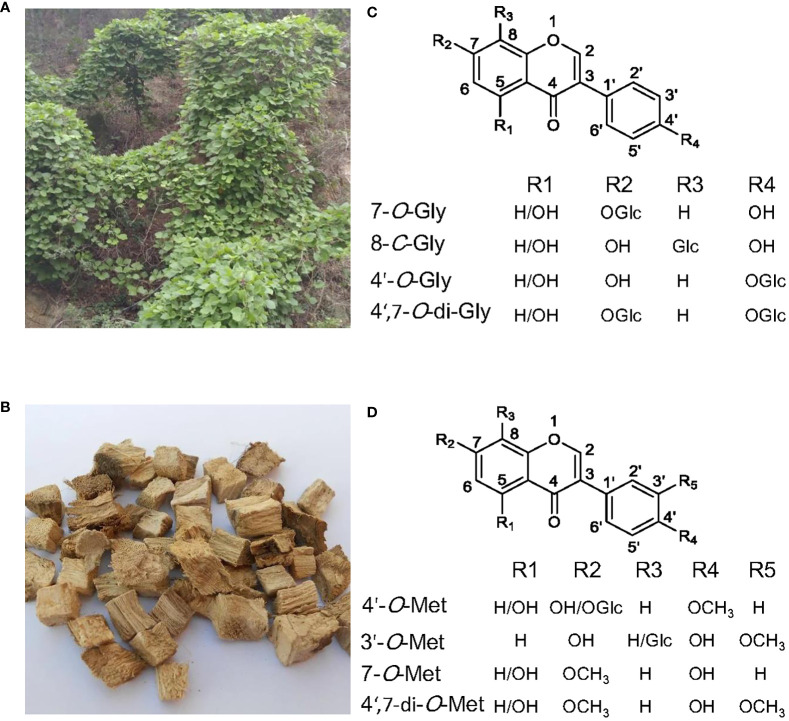
The medicinal plant *Pueraria lobata* and the main glycosylated and methylated isoflavonoids accumulated in it. **(A)** *P. lobata* plant. **(B)** The dried roots of *P. lobata* used in traditional Chinese medicine. **(C)** Structures of the main isoflavone glucosides. **(D)** Structures of the main *O*-methylated isoflavonoids.

Among the 26 *Pueraria* species listed in the plant database (www.theplantlist.org), only three species, *Pueraria lobata* (Willd.) ohwi (**Figure 1A**), *Pueraria thomsonii* Benth, and *Pueraria peduncularis* Benth, have been included into Chinese Pharmacopoeia (Wang et al., 2020). The dried root of *P. lobata* (**Figure 1B**), also called Ge-Gen in China, is used as traditional herb medicine mainly for treating cardiovascular diseases, vascular hypertension, and diabetes (Ehrman et al., 2007; Wong et al., 2011). Modern pharmacological studies have revealed hepatoprotective(Sun et al., 2019), anti-inflammatory (Xi C. et al., 2023), anti-boneloss (Yang et al., 2017), and anti-cancer effects (Ahmad et al., 2020) of *P. lobata* extracts.

Isoflavonoid compounds are considered the main bioactive components of *P. lobata* (Rong et al., 1998). A significant example is puerarin (i.e., daidzein 8-*C*-glucoside), an isoflavone that has been used as a prescribed drug in clinical practice for the treatment of cardiovascular diseases (Zhou et al., 2021). The biosynthetic pathway for the formation of isoflavonoid backbone is predominantly conserved in legumes (Falcone Ferreyra et al., 2012). Subsequent modifications on the isoflavonoid skeleton, such as glycosylation and methylation, result in the difference in isoflavonoid composition between different leguminous species. For instance, puerarin and its glycosylated and/or methylated derivatives (e.g. 3’-methoxy puerarin, 6’’-*O*-xylosylpuerarin and puerarin 4’-*O*-glucoside) are produced only by the species within the *Pueraria* genus (Wang et al., 2020), therefore conferring their unique medicinal value for human. Phytochemical studies revealed glycosylation and methylation as the two major modifications in isoflavonoid metabolism in *P. lobata* (Wang et al., 2020). As a consequence, pharmacological activities of *P. lobata* have focused mainly on the specifically glycosylated or/and methylated isoflavonoids, such as puerarin (Wang et al., 2022) and 3’-methoxy puerarin (Zhao et al., 2007). There is intense interest in identifying the enzymes responsible for the glycosylation and methylation reactions for isoflavonoid metabolism in *P. lobata*. This review considers the recent progress in understanding the biochemistry of the glycosylation and methylation for isoflavonoid metabolism in *P. lobata*.

## Isoflavonoid metabolism in *Pueraria lobata*

Isoflavonoid glycosides from *Pueraria* are mainly the *C*- and *O*-glycosides (Wang et al., 2020). The glycol-conjugation towards *Pueraria* isoflavonoids occurs primarily at the positions of *O*-7, *C*-8, and *O*-4’ (Wang et al., 2020) (**Figure 1C**). Mono-glycosylation at *C*-8 or *O*-7 seems to be prevalent in *P. lobata*, as the most abundant isoflavone glycosides in *P. lobata* include the 7-*O*-glucosides of genistein and daidzein, and the 8-*C*-glucosides of daidzein (i.e., puerarin) (Ohshima et al., 1988; Rong et al., 1998; Wang et al., 2016). *P. lobata* root also accumulates the 4’-*O*-glucosides of puerarin, genistein, and daidzein (Ohshima et al., 1988; Li et al., 2010; Wang et al., 2016), and daidzein 4’,7-*O*-diglucosides (Keung et al., 1996). Glycosylation reaction is enzymatically driven by uridine diphosphate (UDP)-sugar glycosyltransferases (UGTs), and the UGTs involved in plant secondary metabolism usually belong to the family 1 UGTs, which possess the signature PSPG (plant secondary product glycosyltransferase consensus sequence) motif at their *C*-terminal (Vogt and Jones, 2000).

The common sites for methylation of *P. lobata* isoflavonoids are *O*-4’, *O*-3’, and *O*-7 (**Figure 1D**). The majority of methylated isoflavonoids in *P. lobata* are 4’-*O*-methylated isoflavones represented by formononetin (4’-*O*-methyldaidzein) and biochanin A (5-hydroxy formononetin), while the 3’- and 7-*O*-methylated isoflavones are produced in much less amounts (Rong et al., 1998). The 4’- and 7-*O*-methylated isoflavones are also produced in other leguminous plant species, including *Medicago truncatula*, *Glycyrrhiza echinata*, *Medicago sativa*, and *Lotus japonicus* (He et al., 1998; Akashi et al., 2003; Deavours et al., 2006). Interestingly, the occurrence of 3’-*O*-methylated isoflavones seems to be restricted to the *Pueraria* genus. The *O*-methylation reaction is catalyzed by an OMT which transfers a methyl from the donor SAM (S-adenosyl-L-methionine) to a hydroxyl moiety of an acceptor.

## Identification of UGTS and OMTS acting on isoflavonoids in *P. Lobata*

### 8-*C*-glycosyltransferase

**Figure 2:**
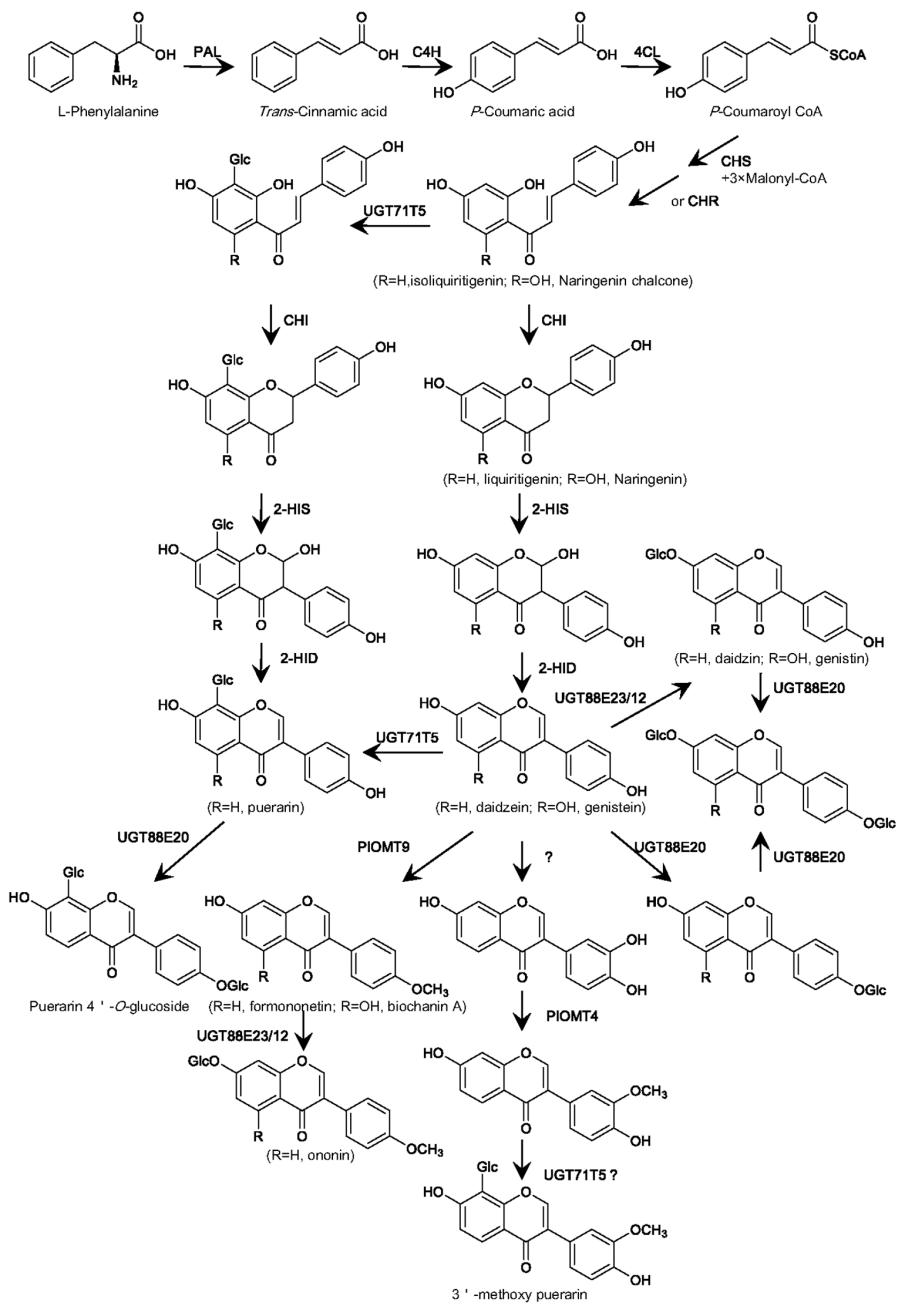
Proposed biosynthesis in *P. lobata* of the main glycosylated and methylated isoflavonoids based on the properties of the *P. lobata* UGTs and OMTs characterized so far. PAL, phenylalanine ammonialyase; C4H, cinnamate 4-hydroxylase; 4CL, 4-coumarate CoA ligase; CHS, chalcone synthase; CHR, chalcone reductase; CHI, chalcone isomerase; 2-HIS, 2-hydroxyisoflavanone synthase; 2-HID, 2-hydroxyisoflavanone dehydratase; UGT, UDP-glucosyltransferase; OMT, *O*-methyltransferase.

The 8-*C*-glycosylation is required for puerarin formation. There is great interest in understanding the biochemical process for the formation of the 8-*C*-glycosyl group in puerarin (Inoue and Fujita, 1977; Wang et al., 2017; Bao et al., 2022). Some data remain contradictory, particularly regarding the step at which the 8-*C*-glucosyl group is introduced (**Figure 2**). Early labeling studies had proposed an upstream intermediate isoliquiritigenin at the chalcone stage, but not daidzein at the isoflavone stage, as an acceptor for the 8-*C*-glycosylation (Inoue and Fujita, 1977). However, an enzyme assay using the *Pueraria* root crude protein provided an implication that the *C*-glucosyl unit in puerarin might be introduced at the isoflavanone stage (He et al., 2011). This assumption is prone to being considered because that a number of flavone *C*-GTs recognize 2-hydroxyflavanone intermediates as their natural substrates (Brazier-Hicks et al., 2009; Nagatomo et al., 2014; Hirade et al., 2015). Nonetheless, Xi et al. revealed that there are no orthologs of 2-hydroxyflavanone *C*-GTs in *P. lobata* (Xi H.T. et al., 2023), indicating that if the *C*-glycosylation for puerarin biosynthesis occurs at the isoflavanone stage, it is probably catalyzed by a phylogenetically distinct UGT. A very recent labeling study provided evidence that both isoliquiritigenin and daidzein could be incorporated into puerarin *in vivo* (Adolfo et al., 2022), suggesting that the 8-*C*-glycosylation can happen at either the chalcone or isoflavone stage, or simultaneously at both levels.

For the first time, a *P. lobata C*-GT (namely PlUGT43), which directly transfers a glucose group to the C-8 position of daidzein leading to puerarin, was molecularly cloned from the root of *P. lobata* by Wang et al. (Wang et al., 2017). Through the *in vitro* assays, PlUGT43 was found to have no or negligible activity with the chalcone intermediate isoliquiritigenin (Wang et al., 2017). However, its isoform (officially named UGT71T5), which shares 99.72% sequence identity with PlUGT43, was recently reported to be capable of catalyzing the *C*-glycosylation activity against both daidzein and isoliquiritigenin (Adolfo et al., 2022). Incubation of the recombinant PlUGT43 or UGT71T5 with 2-hydroxyisoflavanone did not generate a product matching the 2-hydroxyisoflavanone *C*-glycoside (Wang et al., 2017; Adolfo et al., 2022), indicating that they had no *C*-glycosylation activity with 2-hydroxyisoflavanone. The RNAi-mediated down-regulation of UGT71T5 caused a strong reduction in the levels of puerarin in *P. lobata* hairy roots (Adolfo et al., 2022), confirming that PlUGT43 (UGT71T5) functions as a *C*-GT at least partially for puerarin biosynthesis in *P. lobata*. Interestingly, when the 2-HIS (2-hydroxyisoflavanone synthase; see its place in the pathway in **Figure 2**), which is the entry enzyme catalyzing the formation of isoflavonoid backbone (Steele et al., 1999; Jung et al., 2000), was down-regulated in *P. lobata* hairy roots, the potential *C*-glycosides of isoliquiritigenin and/or liquiritigenin significantly accumulated, when compared to that in the control roots (Adolfo et al., 2022). This data clearly supports that introduction of the *C*-glucosyl group can take place at the chalcone stage and then the 2-HIS would be able to accommodate the *C*-glycosides as substrates (**Figure 2**). Recently, another variant of PlUGT43, designated PlCGT, was identified from *P. lobata* by Ye et al. (Bao et al., 2022). PlCGT shows 97.01% amino acid identity with PlUGT43, and this variant essentially catalyzes the same *C*-glycosylation activity as PlUGT43 (Bao et al., 2022). An ortholog of PlUGT43, named PtUGT8, was also isolated from *P. thomsonii* species (Duan et al., 2022). Despite exhibiting a high sequence identity (96.52%) to PlUGT43, PtUGT8 was shown as primarily having a 7-*O*-glucosylation activity toward isoflavones whereas not catalyzing the 8-*C*-glycosylation reaction as does by PlUGT43 (Duan et al., 2022). In view of a previous finding that *O*- and *C*-GT can be easily shifted by changing only a few amino acids (Gutmann and Nidetzky, 2012), a subtle difference in the active sites of PtUGT8 and PlUGT43 may plausibly account for this discrepancy.

Taken together, combination of the published data from the *in vitro* assays using the recombinant UGTs, the *in vivo* labeling experiments, and the transgenic studies of *P. lobata* hairy roots strongly supports that during puerarin biosynthesis, the *C*-glucosylation reaction takes place most likely at either the chalcone or isoflavone stage, or both.

### 7-*O*-glycosyltransferase

The 7-*O*-glycosylation is common for isoflavonoid metabolism in leguminous plant species. In the 1980s, a relatively pure protein bearing the isoflavone 7-*O*-glucosylation activity was first purified from *Cicer arietinum* L (Koster and Barz, 1981). Later, genes encoding isoflavone 7-*O*-glucosyltransferases were isolated from the cell suspension cultures of *Glycyrrhiza echinata* (Nagashima et al., 2004), the roots (Noguchi et al., 2007) and seeds (Dhaubhadel et al., 2008) of *Glycine max*.

A total of six *P. lobata* UGTs, named PlUGT1(official UGT designation UGT88E12), PlUGT13(UGT88H1), PlUGT4(UGT72Y3), PlUGT15(UGT88E23), PlUGT57(UGT84F7) and UGT88A40, had been characterized by *in vitro* assays as having an isoflavone 7-*O*-glucosylation activity on daidzein and genistein (Li et al., 2014; Wang et al., 2019; Adolfo et al., 2022). Analysis of the substrate conversion rate and enzymatic kinetic parameter revealed that both PlUGT1 and PlUGT15 are highly specific for isoflavones with no or little activity against other acceptors, including chalcones, flavanones, flavones, and flavonols (Li et al., 2014; Wang et al., 2019). The PlUGT13, PlUGT4, or UGT88A40 can accept relatively broad substrates and glycosylates substrates at different positions (Li et al., 2014; Adolfo et al., 2022). Although PlUGT57 also shows a strict substrate preference for isoflavone aglycones, its catalytic efficiency (K_cat_/K_m_; 2.10 × 10^3^ M^-1^s^-1^) toward daidzein, when UDP-glucose is used as a sugar donor, is about 20-fold lower than UGT88E12 (3.79 × 10^4^ M^-1^s^-1^), and 80-fold lower than UGT88E23(1.75 × 10^5^ M^-1^s^-1^) (Wang et al., 2019). Phylogenetic analysis (Li et al., 2014; Wang et al., 2019) revealed that PlUGT1 and PlUGT15 showed a close relationship with a *G. max* 7-*O*-UGT GmIF7GT (UGT88E3) that shows a substrate preference for isoflavones (Noguchi et al., 2007). Therefore, members of the UGT88E family are believed to truly contribute to the 7-*O*-glycosylation in isoflavonoid metabolism in *P. lobata*.

### 4’-*O*-glycosyltransferase

The presence of 4’-*O*-glucosides of daidzein, genistein, and puerarin (Ohshima et al., 1988; Fang et al., 2006), and 4’,7-*O*-diglucoside of daidzein (Zhang et al., 2013) in *P. lobata* tissues suggests the occurrence of UGTs specific for 4’-*O*-glycosylating these compounds.

One full-length cDNA encoding PlUGT2 (officially assigned as UGT88E20) was identified and cloned from *P. lobata* using an RNA-sequencing approach (Wang et al., 2016). Tissue-specific expression analysis indicated that the transcript of PlUGT2 was higher in roots relative to stems and leaves. Phylogenetic analysis (Wang et al., 2016) showed that PlUGT2 was grouped into the same clade with GmUGT1 and GmUGT7 from *Glycine max*, which are the flavone 4’-*O*-UGTs (Funaki et al., 2015). The purified protein of PlUGT2 could catalyze either *O*-4’- or *O*-7-glucosylation of genistein, daidzein, liquiritigenin, and naringenin, yielding their mono-4’-*O*- or 7-*O*-glucosides. Interestingly, PlUGT2 consecutively glycosylates these mono-glucosides to di-glucosides with both *O*-4’ and *O*-7 being glucosylated (Wang et al., 2016). In comparison with the mono-glycosylation, the di-glycosylation activity catalyzed by PlUGT2 is much lower, consistent with the fact that the 4’,7-*O*-di-glucosides are produced at extremely low levels in *P. lobata* tissues(Zhang et al., 2013). PlUGT2 is the first 4’-*O*-glucosyltransferase identified from *P. lobata*. Recently, Adolfo et al. (Adolfo et al., 2022) reported another *P. lobata* UGT, named UGT73C42, which catalyzes either 4’- or 7-*O*-glucosylation of various polyphenolic compounds, including chalcones, flavones, and isoflavones.

PlUGT2 could also catalyze 4’- or 7-*O*-glucosylation of puerarin (Wang et al., 2016), which is currently a clinical drug for curing cardiovascular diseases (Zhou et al., 2021). Although puerarin is currently a prescribed drug, its low water solubility is still a serious drawback in clinical applications (Wang et al., 2012; Chen et al., 2021). Glycosylation is an efficient way to increase water solubility(Liu et al., 2016), thus, the identification of PlUGT2 would provide such an opportunity.

### 3’-*O*-methyltransferase

Many isoflavonoids are *O*-methylated with the methoxy residue improving their biological activities by increasing liposolubility (Wen et al., 2017). *P. lobata* accumulates 3’-methoxy-derivatives of isoflavones, including 3’-methoxydaidzein, 3’-methoxydaidzin, 3’-methoxypuerarin, and 3’-methoxyformononetin (Rong et al., 1998; Li et al., 2016b; Wang et al., 2020). Relative to the puerarin itself, its derivative 3’-methoxypuerarin exhibited better protective effects on cerebral ischemic-reperfusion injury in rats (Zhao et al., 2007).

One OMT, designated PlOMT4, was cloned (Li et al., 2016b) from *P. lobata* based on the *P. lobata* transcriptome database (Wang et al., 2015). Tissue-specific expression analysis revealed that PlOMT4 was expressed most highly in roots, and its transcript was up-regulated by MeJA (Li et al., 2016b). PlOMT4 was found to have the activity of methylating 3’-hydroxy daidzein to form 3’-methoxy-daidzein (Li et al., 2016b). PlOMT4 has no activity with the isoflavonoid substrates with free hydroxyl groups at either C7 or C4’ (Li et al., 2016b), suggesting the methylation activity of PlOMT4 is region-specific. In addition, PlOMT4 is inactive with 3’-hydroxy puerarin (Li et al., 2016b), indicating that the 8-*C*-glucosylation of 3’-hydroxy daidzein prevents methylation at the 3’-position, and thereby the 3’-methylation should take place prior to the 8-*C*-glycosylation during 3’-methoxy-puerarin biosynthesis. PlOMT4 seems to be the only isoflavone specific 3’-*O*-methyltransferase so far identified from plant species.

### 4’- *O*-methyltransferase

In the early 1970s, scientists began a search for the isoflavone 4’-*O*-methyltransferase (I4’OMT) from plants. At a protein level, a methyltransferase, which catalyses the 4’-*O*-methylation of the isoflavone daidzein, was purified from *Cicer arietinum* L (Wengenmayer et al., 1974), indicating that the 4’-*O*-methylation for biosynthesis of 4’-*O*-methylated isoflavonoids can take place at the isoflavone stage. However, in alfalfa (*Medicago sativa* L.) seedlings, radiolabeled daidzein is not incorporated into 4’-*O*-methylated isoflavonoids (Dewick and Martin, 1979). Paradoxically, biosynthesis of the 4’-*O*-methylated isoflavonoids in alfalfa suspension cells strongly correlates with the isoflavone 7-*O*-methyltransferase (I7OMT) activity (Edwards and Dixon, 1991; He et al., 1998), and over-expression of the I7OMT led to enhanced levels of 4’-*O*-methylated isoflavonoids in the elicited alfalfa leaves(He and Dixon, 2000). In the elicited alfalfa leaves, the operationally soluble I7OMT re-locates to the endoplasmic reticulum where the 2-HIS naturally resides, leading to an interesting hypothesis that the association with other isoflavonoid pathway enzymes may change the region-specificity of I7OMT from the 7- to 4’-position *in vivo* (Liu and Dixon, 2001). On the other hand, an enzyme assay with the *Glycyrrhiza echinata* cell-free extract demonstrated that the 4’-*O*-methylation occurs at the level of 2,7,4’-trihydroxyisoflavanone (Akashi et al., 2000). This is supported by the molecular cloning and characterization of cDNAs encoding the 2,7, 4’-tri-hydroxyisoflavanone 4’-*O*-methyltransferase (HI4’OMT) from *Glycyrrhiza echinata* (Akashi et al., 2003), and *Medicago truncatula* (Deavours et al., 2006). The isoflavone daidzein could not be converted by HI4’OMT, and it only recognizes 2-trihydroxy-isoflavanone as the direct methyl acceptor (Akashi et al., 2003), suggesting that HI4’OMT catalyzes the 4’-*O*-methylation reaction only at the isoflavanone stage. Therefore, the history leading to the finding of isoflavonoid 4’-*O*-methyltransferases demonstrates two alternative pathways likely involved: one is the simplest 4’-*O*-methylation occurring at the isoflavone stage, and the other is the reaction performed at the level of 2-hydroxyisoflavanones. From *P. lobata*, Li et al. identified a novel isoflavone 4’-*O*-methyltransferase (designated PlOMT9) that is capable of directly 4’-*O*-methylating isoflavones (Li et al., 2016a). Because that PlOMT9 shows the highest degree of amino acid identity with the isoflavone 7-*O*-methyltransferases (I7OMTs), PlOMT9 was initially presumed as an I7OMT. However, yeast cells expressing PlOMT9 efficiently performed the 4’-*O*-methylation of daidzein, genistein, prunetin, and isoformononetin (Li et al., 2016a), demonstrating that PlOMT9 functions actually as a I4’OMT. The I4’OMT activity catalyzed by PlOMT9 was further confirmed by *in vitro* assays using the purified recombinant PlOMT9 (Li et al., 2016a). Moreover, the recombinant PlOMT9 was not active with 2,7,4’-trihydroxy-isoflavanone, which is the natural substrate of HI4’OMT (Akashi et al., 2003). In addition to the main I4’OMT activity, PlOMT9 retains an extremely low 7-*O*-methylation activity, such as *O*-methylating daidzein at C7 position to yield trace amounts of isoformononetin. Over-expression of *PlOMT9* in *Glycine max* hairy roots increased the levels of formononetin and ononin (formononetin 7-*O*-glucoside) by 111.2% and 940.9%, respectively, in comparison with the controls. *P. lobata* contains a HI4’OMT-like enzyme (Li et al., 2016a), which shares 73% amino acid identity with the HI4’OMT from *G. echinata* (Akashi et al., 2003), but it is inactive either with 2,7,4’-trihydroxy-isoflavanone or the isoflavone daidzein (Li et al., 2016a).

## Conclusion and prospects

In summary, utilizing the transcriptomic analysis, in combination with *in vitro* biochemical analysis of recombinant protein, provides a strong basis for understanding the biosynthetic mechanism of glycosylation and methylation of isoflavonoids in *P. lobata* (**Figure 2**). For the *O*-glycosylation of isoflavonoids in *P. lobata*, either 7-*O*- or 4’-*O*-glycosyltransferase protein is from members of the UGT88E subgroup. For the *C*-glycosylation of isoflavonoids, PlUGT43 (official designated UGT71T5) is the only isoflavone *C*-GT identified from plants so far. For the *O*-methylation of isoflavonoids in *P. lobata*, both 3’- and 4’-*O*-methyltransferases perform the methylation reactions at the isoflavone stage, directly utilizing isoflavones as the best acceptors.

Of particular value among the isoflavonoids are puerarin and its derivatives, which are produced exclusively in *Pueraria* species. Puerarin has been established as a clinical drug to deal with cardiovascular diseases (Wang et al., 2022). By expressing the *PlUGT43*, in combination with other pathway genes, the production of puerarin directly from glucose could be achieved in yeast at a concentration of 72.8 mg/L (Liu et al., 2021). The poor water solubility of puerarin is still a challenge in narrowing its treatment window in clinical usage (Liu et al., 2016). Considering that glycosylation is the most effective way to increase water solubility of small molecules (Li et al., 2004), the PlUGT2, which is capable of glucosylating puerarin, would provide an excellent template for further designing novel enzymes to increase the water solubility of puerarin.

## Author contributions

CL: Writing – original draft. YZ: Funding acquisition, Writing – review & editing.
